# Metabolic Fingerprinting of Seminal Plasma from Non-obstructive Azoospermia Patients: Positive Versus Negative Sperm Retrieval

**Published:** 2018

**Authors:** Kambiz Gilany, Naser Jafarzadeh, Ahmad Mani-Varnosfaderani, Arash Minai-Tehrani, Mohammed Reza Sadeghi, Mahsa Darbandi, Sara Darbandi, Mehdi Amini, Babak Arjmand, Zhamak Pahlevanzadeh

**Affiliations:** 1- Reproductive Biotechnology Research Center, Avicenna Research Institute, ACECR, Tehran, Iran; 2- Metabolomics and Genomics Research Center, Endocrinology and Metabolism Molecular Cellular Sciences Institute, Tehran University of Medical Sciences, Tehran, Iran; 3- Department of Medical Physics, Tarbiat Modares University, Tehran, Iran; 4- Chemometrics and Chemoinformatics Laboratory, Department of Chemistry, Faculty of Sciences, Tarbiat Modares University, Tehran, Iran; 5- Nanobiotechnology Research Center, Avicenna Research Institute, ACECR, Tehran, Iran; 6- Cell Therapy and Regenerative Medicine Research Center, Endocrinology and Metabolism Molecular Cellular Sciences Institute, Tehran University of Medical Sciences, Tehran, Iran; 7- Metabolomics and Genomics Research Center, Endocrinology and Metabolism Molecular Cellular Sciences Institute, Tehran University of Medical Sciences, Tehran, Iran

**Keywords:** Male infertility, Metabolic fingerprinting, Non-obstructive azoospermia, Seminal plasma, Testicular sperm extraction

## Abstract

**Background:**

Non-obstructive azoospermia (NOA) occurs in approximately 10% of infertile men. Retrieval of the spermatozoa from the testicle of NOA patients is an invasive approach. Seminal plasma is an excellent source for exploring to find the biomarkers for presence of spermatozoa in testicular tissue. The present discovery phase study aimed to use metabolic fingerprinting to detect spermatogenesis from seminal plasma in NOA patients as a non-invasive method.

**Methods:**

In this study, 20 men with NOA were identified based on histological analysis who had their first testicular biopsy in 2015 at Avicenna Fertility Center, Tehran, Iran. They were divided into two groups, a positive testicular sperm extraction (TESE(+)) and a negative testicular sperm extraction (TESE(−)). Seminal plasma of NOA patients was collected before they underwent testicular sperm extraction (TESE) operation. The metabolomic fingerprinting was evaluated by Raman spectrometer. Principal component analysis (PCA) and an unsupervised statistical method, was used to detect outliers and find the structure of the data. The PCA was analyzed by MATLAB software.

**Results:**

Metabolic fingerprinting of seminal plasma from NOA showed that TESE (+) versus TESE(−) patients were classified by PCA. Furthermore, a possible subdivision of TESE(−) group was observed. Additionally, TESE(−) patients were in extreme oxidative imbalance compared to TESE(+) patients.

**Conclusion:**

Metabolic fingerprinting of seminal plasma can be considered as a breakthrough, an easy and cheap method for prediction presence of spermatogenesis in NOA.

## Introduction

Male factor contributes to the infertility of half of the infertile couples (1). Approximately 6–10% of the infertile men ejaculate lacks spermatozoa due to testicular failure; a condition called non-obstructive azoospermia (NOA) (2). However, a possible small number of spermatozoa can be found in some NOA patients, which can be used for intracytoplasmic sperm injection (ICSI) (3, 4). Finding the spermatozoa from the testicle of NOA patients has been reported as a big challenge in clinics (5). Several predictors have been suggested for retrieval of spermatozoa including history of ejaculated sperm, serum hormones, testis volume and testis biopsy histology (6–8). All these parameters have been shown to be poor predictors of spermatozoa existence in NOA patients. One of the most successful sperm retrieval methods is invasive testicular sperm extraction (TESE). The mentioned method present about 60–65% success in spermatozoa finding (3). Thus, the reproductive medicine is needed as a more sensitive and accurate technique of spermatozoa retrieval from NOA patients.

State-of-the-art metabolomics technology has been developing to become a key tool for realizing and diagnosis of male infertility (9–11). Metabolomics is defined as a study of the metabolites (small molecules less than 1500 *Da*). Metabolome is dynamic and is close to the phenotype. Metabolome covers a wide range of molecules, *e.g*. amino acids, organic acids and fatty acids (12). Currently, 40,000 human metabolites can be found in the most recent version of the Human Metabolome Database (13). Since the definition of metabolome, almost 20 years ago, different terms related to metabolite quantification and quality measurement have been defined, including metabolome mapping, metabolic fingerprinting, metabolic profiling, metabolic footprinting, and metabolic target analysis to untargeted metabolic profiling (10). The metabolic fingerprinting has successfully been shown using seminal plasma of asthenozoospermia and idiopathic infertile men that can be used as an excellent diagnosis tool (14, 15).

Seminal plasma is an excellent source of biological material to explore for finding the cause of male infertility (16). For an unknown reason, it has not received much attention in metabolomics studies of male infertility. A search in the Pubmed by the keyword “seminal plasma” and “metabolomics” shows only 19 hits. Furthermore, a few researchers have applied metabolomics technology to improve a diagnostic device for recognition or finding a possible biomarker of spermatozoa in NOA patients. To the best of our knowledge, Lynch et al. investigated, for the first time, metabolic profiling of seminal plasma of azoospermia using nuclear magnetic resonance spectroscopy (^1^H-NMR) in 1994. They showed the potential of application of ^1^H-NMR analysis for seminal plasma composition. Hamamah et al. did the second study which showed the application of magnetic resonance spectroscopy (^1^H-MRS) to differentiate a metabolite in seminal plasma of obstructive azoospermia. Aaronson et al. used the ^1^H-MRS instrument on the frozen testicular tissue of NOA men as a biological material to determine a metabolic signature for finding spermatozoa. Furthermore, recently 36 potential biomarkers were identified by metabolic profiling of seminal plasma in NOA patients using gas chromatography-mass spectrometry (GCMS) instrument (17–20). However, all the mentioned studies are not straight-forward for clinical laboratory use and need proper expertise to handle the instrumentations.

The easiest and fastest method for metabolome study is metabolic fingerprinting. The instrument used in this technique is a typical optical spectrometer *e.g*. Raman Spectroscopy. The advantages of optical spectroscopy usage are the sample preparation and handling compared to other related instruments (15).

To the best of our knowledge, no metabolic fingerprinting of seminal plasma has been reported in NOA with testicular sperm extraction positive (TESE(+)) or testicular sperm extraction negative (TESE(−)) patients. In this study, a metabolic fingerprinting was reported that can be used as a potential diagnosis tool for detection of spermatogenesis.

## Methods

### Sample selection:

The samples were collected from NOA men attended to Avicenna Fertility Center (Tehran, Iran) for infertility treatment. The ejaculate of NOA patients was collected before TESE operation and kept at −80*°C* for further analysis. The positive and negative results of TESE of collected samples were confirmed after the operation. The surgeon removed 8–10 biopsies from the testes in order to observe the presence of spermatozoa following tissue dissection and searching under microscope. Totally, 10 non-obstructive azoospermia testicular sperm extraction positive (NOA TESE(+)) and 10 non-obstructive azoospermia testicular sperm extraction negative (NOA TESE(−)) patients were subjected to analysis in this study. Additionally, 15 samples of human seminal plasma of voluntary fertile individuals were used as control. Our investigation has been approved by the ethics committee of Avicenna Research Institute, Tehran, Iran (Code: 910107-023).

### Metabolome extraction:

Metabolome of seminal plasma was extracted as described before (15, 20). Briefly, 400 *μl* seminal plasma was mixed with 500 *μl* cold methanol/water. The mixture was placed on ice for 20 *min*. The mixture was then centrifuged for 8 *min* at 6,000 *rpm*. The upper phase was used for Raman Spectroscopy.

### Raman Spectrometer:

All spectra were collected by Almega Thermo Nicolet Dispersive with the following specific parameter: the spectral range: 100–4,200 *cm*^−1^; Laser: second harmonic at 532 *nm* of a Nd:YLF laser; resolution: 4 *cm*^−1^; and laser power: 30 *mW*. Next, 30 scans per spectra were taken. Each sample was analyzed in triplicate. In total, 62 spectra were collected for chemometrics analysis.

### ROS measurement:

ROS measurement was carried out according to the manual instruction as described by Agarwal et al. Briefly, the freshly ejaculated and completely liquefied semen was centrifuged at 3400 *rpm* for 5 *min*. Human seminal plasma was prepared. ROS levels were measured by chemiluminescence assay using luminol (5-amino-2, 3- dihydro-1, 4-phthalazinedione) and results were expressed as relative light units (RLU)/sec (21).

### Statistical analysis:

A multivariate analysis of the dataset obtained from Raman spectra of the human seminal plasma samples was performed by MATLAB. Principal component analysis (PCA) as an unsupervised statistical method for detecting the outliers and finding the structure of the data was used in this study. All computer programs involved in this study were coded in-house with MATLAB software (version 7.012.635, R2011a). The calculations were implemented on a desktop computer with “Windows 7 Pro” as the operating system, Intel(R) Core(TM) core i7 CPU and 8GB of RAM memory.

## Results

### Metabolomic fingerprinting:

**Figure 1.**
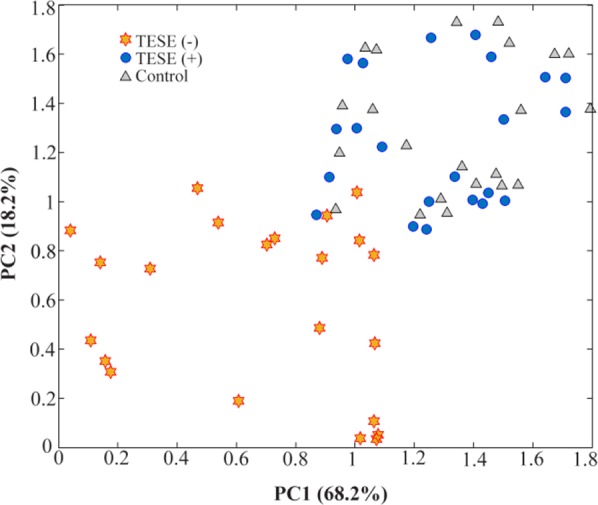
The results of principle component (PC) analysis of the data matrix of fertile (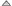), TESE (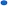) versus TESE(−) (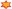). Each patient was analyzed in triplicate

In this study, metabolic fingerprinting of seminal plasma from NOA patients of TESE(+) versus TESE(−) was used to detect spermatogenesis. Seminal plasma of twenty NOA patients was analyzed in triplicate by Raman spectrometer. The chemometrics techniques used in this study were data matrix by using multivariate pattern recognition such as principal component analysis. Our results showed that TESE (+) and TESE(−) patients could be classified in separate groups. Furthermore, an overlap between fertile and TESE(+) group was observed (Figure 1).

**Figure 2.**
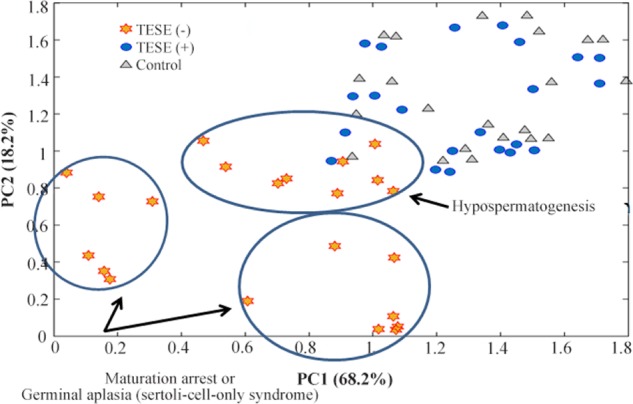
PCA analysis of Raman spectra gives a possible subdivision of TESET(−) groups. TESE(−) group are subdivided to hypospermatogenesis, maturation arrest and germinal aplasia (Sertoli-cell-only syndrome) based on the position in the graph

Additionally, metabolic fingerprinting of seminal plasma of TESE(−) showed that it is possible to subdivide the TESE(−) in 3 different groups (Figure 2). Since there was no information regarding TESE(−) subdivision, it was suggested that the closest classified group to TESE(+) and fertile group was hypospermatogenesis. The other two groups were maturation arrest or germinal aplasia (Sertoli-cell-only syndrome) (22). Collection of each Raman spectra took 30 seconds.

Our results clearly showed that the use of the seminal plasma metabolome can be a perfect source for detection of spermatogenesis.

### Oxidative imbalance:

**Figure 3.**
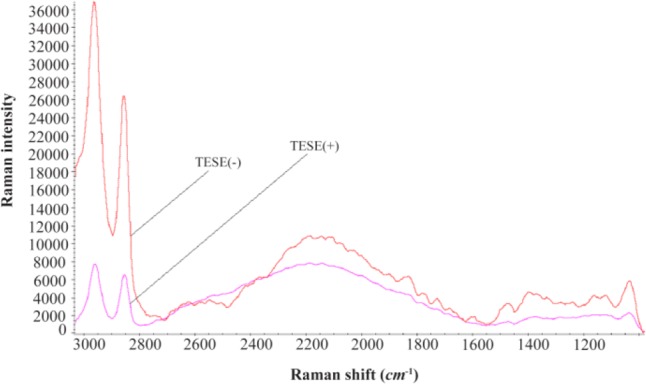
The average spectra of triplicated analysis of 10 TESE(+) versus 10 TESE(−) patients using Raman spectra. The –CH functional group (2800–3000 *cm*^−1^) is biomarker of oxidative imbalance

It has been suggested for a while that oxidative imbalance might be a cause for male infertility, since it can damage spermatozoa (23, 24). Furthermore, it has been shown that there is oxidative imbalance in the azoospermia patients by the terminal uridine deoxynucleotidyl transferase dUTP nick-end labelling (TUNEL) assay (25). Raman spectrometer is a semi-quantitative instrument. It was previously shown that there is an oxidative imbalance in idiopathic infertile men by metabolic fingerprinting of seminal plasma. The –CH functional group (2800–3000 *cm*^−1^) in Raman spectra was a biomarker of oxidative stress (14, 24). Figure 3 showed that TESE(−) patients were in extreme oxidative imbalance compared to TESE(+).

Here, an extremely oxidative imbalance was indicated in TESE(−) compared to TESE(+).

In order to analyze extreme variations of oxidative imbalance in these groups, ROS level was measured in 3 TESE(+) and 5 TESE(−) patients. Our primary results showed, on average, the ROS level was 901 RLU for TESE(+) and 1968 RLU for TESE(−) patients.

## Discussion

There have been only a limited number of studies based on metabolomics technology for the detection of spermatogenesis in NOA patients. To the best of our knowledge, there are only 3 studies which have used seminal plasma for the detection of spermatogenesis in NOA patients to find potential biomarkers. We conducted the latest study to find potential biomarkers for detection of spermatogenesis in NOA patients. In our finding, we integrated proteomics data and metabolomics results suggesting the polyol pathway to be considered for finding the potential biomarkers in NOA patients. However, the applied metabolomics approach cannot be setup easily. It needs a professional expert to use the specified instrument such as gas-chromatography mass spectrometry (GCMS). Therefore, our new metabolomics approach, metabolic fingerprinting is easier to be applied for detection of spermatogenesis in NOA patients. Furthermore, it is simple to create a database of metabolic fingerprinting profile for fertile men compared to NOA patients by Raman spectrometer in order to detect spermatogenesis.

## Conclusion

The metabolomics approaches can be divided based on how fast and reliable they are for measurement. There are a few metabolomics studies available on the NOA. However, these studies do not introduce a good approach for diagnosis and predication of spermatogenesis.

To the best of our knowledge, this is the first metabolic fingerprinting analysis indicating that Raman spectroscopy combined with chemometrics can recognize major alterations at the metabolome level in the seminal plasma of NOA from TESE(+) versus TESE(−) patients. Here, it was shown that TESE(+) versus TESE(−) patients can be classified in two different groups. Furthermore, a subdivision in TESE(−) patients could be observed. It was indicated that metabolic fingerprinting of seminal plasma can be developed as a non-invasive method for detecting spermatogenesis. Additionally, an extremely oxidative imbalance in TESE(−) patients was observed. Additionally, to the best of our knowledge this study is the first study showing the oxidative imbalance between TESE(+) and TESE(−). In this study, it was suggested that an easy setup of ROS measurement can be used as a double test followed by metabolic fingerprinting in detection of spermatogenesis. It is suggested that a first line defense against ROS activity may be considered (26, 27). Therefore, an antioxidant therapy could improve spermatogenesis in NOA patients.

In this study, we hypothesize an easy setup for ROS measurement that can be used as an alternative test followed by metabolic fingerprinting in detection of spermatogenesis. Additionally, we recommend that an antioxidant therapy can improve spermatogenesis in NOA patients. However, these suggestions need to be confirmed.
